# A mutation in the filamin c gene causes myofibrillar myopathy with lower motor neuron syndrome: a case report

**DOI:** 10.1186/s12883-019-1410-7

**Published:** 2019-08-17

**Authors:** Juanjuan Chen, Jun Wu, Chunxi Han, Yao Li, Yuzu Guo, Xiaoxin Tong

**Affiliations:** 1 Department of Neurology, Peking University Shenzhen Hospital, Lianhua Road 1120, Futian District, Shenzhen, 518036 China; 2 Department of Neurology, Shenzhen Children’s Hospital, Shenzhen, China

**Keywords:** Filamin C gene, Myofibrillar myopathy, Distal myopathy, Missense mutation, Lower motor neuron

## Abstract

**Background:**

Myofibrillar myopathies (MFMs) are a genetically heterogeneous group of muscle disorders**.** Mutations in the filamin C gene (*FLNC*) have previously been identified in patients with MFM. The phenotypes of FLNC-related MFM are heterogeneous.

**Case presentation:**

The patient was a 37-year-old male who first experienced weakness in the distal muscles of his hand, which eventually spread to the lower limbs and proximal muscles. Serum creatine kinase levels were moderately elevated. Obvious neuropathic changes in the electromyographic exam and edema changes in lower distal limb magnetic resonance imaging were observed. Histopathological examination revealed the presence of abnormal protein aggregates and angular atrophy in some muscle fibers. Ultrastructural analysis showed inordinate myofibrillar structures and dissolved myofilaments. DNA sequencing analysis detected a heterozygous missense mutation (c.7123G > A, p.V2375I) in the immunoglobulin (Ig)-like domain 21 of *FLNC*.

**Conclusions:**

*FLNC* mutation c.7123G > A, p.V2375I in the immunoglobulin (Ig)-like domain 21 can be associated with distal myopathy with typical MFM features and lower motor neuron syndrome. Although electromyographic examination of our patient showed obvious neuropathic changes, MFM could not be excluded. Therefore, genetic testing is necessary to make an accurate diagnosis.

**Electronic supplementary material:**

The online version of this article (10.1186/s12883-019-1410-7) contains supplementary material, which is available to authorized users.

## Background

Myofibrillar myopathies (MFMs) are a type of inherited muscular disease with variable clinical presentations. The pathological characteristics include focal dissolution of myofibrils and abnormal accumulation of degradation proteins. Mutations in several genes have been associated with MFM, including *DES*, *CRYAB, MYOT, LDBS*, and *FLNC*, which encode Z-disk proteins [1].

Filamin C (FLNC) is an actin cross-linking protein and also one of the largest Z-disk proteins in cardiac and skeletal muscle. Its main function is to connect muscle cells to the extracellular matrix and be involved in related signaling pathways [2]. The filamin C-related myopathy was first described in 2005. The patients from a large German family with the nonsense mutation (c.G8130A, p.W2710X) in the FLNC immunoglobulin (Ig) like domain 7 had predominant limb-girdle muscle weakness [3, 4]. However, the phenotypes of FLNC-related MFM are heterogeneous. Some patients with mutations in the actin-binding domains or Ig-like domain 15 present with weakness in their distal muscles and show no cardiomyopathy or typical pathology. Other patients have peripheral nerve damage, ataxia, and cerebellar atrophy [5].

Lower motor neuron (LMN) syndrome affects anterior horn cells or peripheral motor nerves. Patients may have focal muscle atrophy, weakness, fasciculations, and hyporeflexia, but no sensory problems. This syndrome is seen in some hereditary muscular diseases such as spinal muscular atrophy (SMA) and some degenerative diseases [6]. However, there are no reports of FLNC-related myopathy associated with LMN syndrome. Here, we describe the clinical, histological, and muscle magnetic resonance imaging (MRI) data of a Chinese patient with a complex phenotype characterized by pathologically confirmed MFM associated with LMN syndrome. The molecular culprit in this case was a 7123G > A, p.V2375I *FLNC* mutation.

## Case presentation

**Fig. 1:**
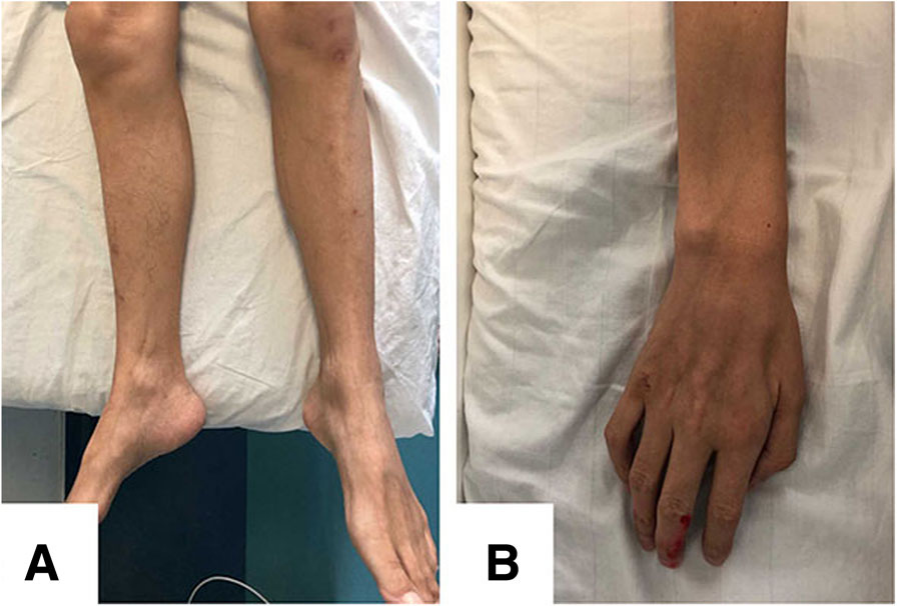
Clinical pattern of the patient. Distal limb weakness affecting both upper and lower extremities, with conspicuous muscle atrophy were seen (**a**: gastrocnemius and anterior tibial muscles, **b**: interosseus muscles of the hand)

The patient was a 37-year-old male from a non-consanguineous Chinese family. Since the age of 35, he had experienced progressive weakness of his hands and a reduction of grip strength, especially in his right hand. Six months later, muscle atrophy and muscle fibrillation were noticed in his hands, and he was unable to hold things or to write. One year later, he experienced weakness in his lower extremities with no sensory disturbance. He currently experiences difficulty in climbing the stairs and standing up from a squatting position, is unable to lift his foot upward, and trips over easily. Physical examination revealed that the cranial nerves were normal, and that orolingual fasciculations and atrophy were absent. The neck flexion strength was 5 (MRC muscle scale, grades 0–5). The muscle strength of both sides of the body was as follows: triceps and biceps 3/3, forearm flexors 2/2, intrinsic hand muscles 1/1, iliopsoas muscles 4/4, quadriceps muscles 3/3, tibialis anterior and gastrocnemius muscles 2/2. Deep tendon reflexes were absent. There was no sensory abnormality or coordination difficulty of any of the limbs. Atrophy was seen in most of the muscles, especially the interosseous muscles of the hands, bilateral gastrocnemius and anterior tibial muscles (Fig. 1). Muscle fibrillation was observed in the biceps and quadriceps muscles.

**Fig. 2:**
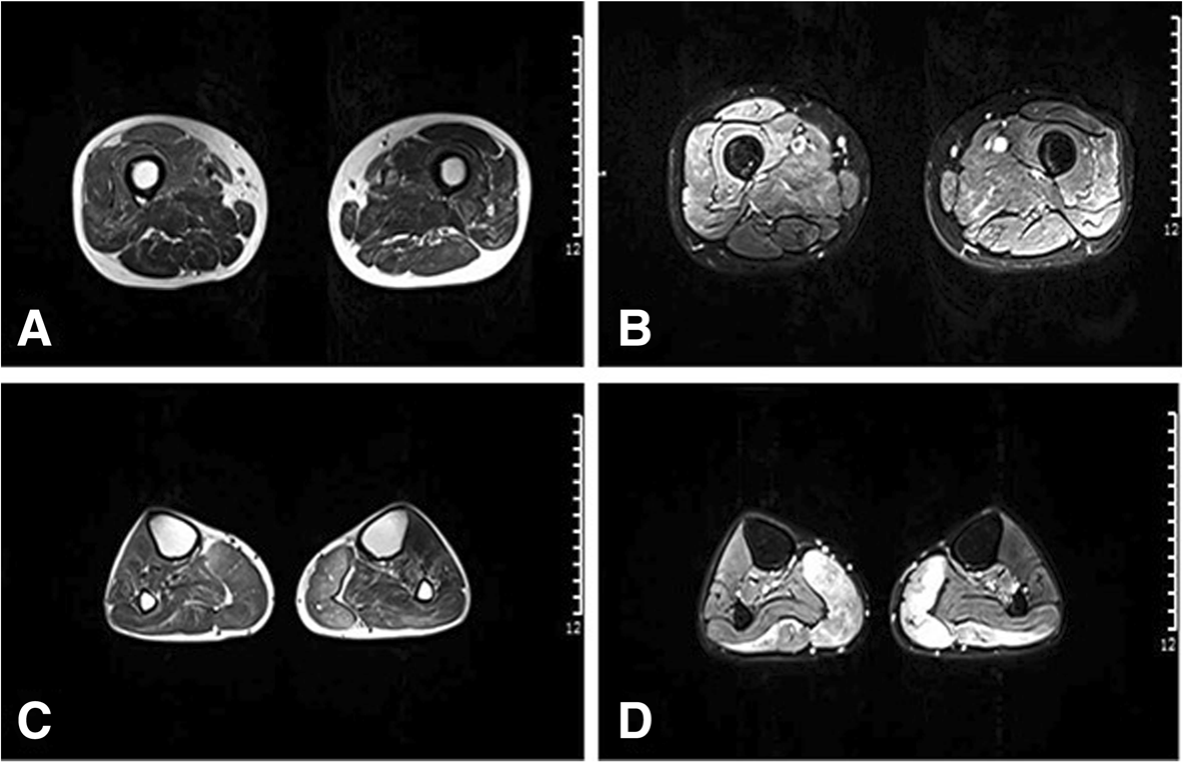
Transverse T2-weighted and TIRM sequence muscle magnetic resonance images. A slightly increased signal was detected in the posterior compartment of the thigh, in the semimembranosus and semitendinosus muscles (**a**: T2-weighted, **b**: TIRM). A highly increased signal intensity in TIRM sequences was detected at the level of the lower leg, indicating obvious muscular edema (**d**). A mild increase in the signal intensity was observed in the T2 sequence of soleus and tibialis anterior muscles, indicating fat replacement (**c**)

The patient’s serum level of creatine kinase was 668 U/L (normal range, 50–310 U/L). Extractable nuclear antigens were negative, and serum sex hormone levels were normal. Peripheral neuropathy antibodies such as GM1-antibody and GQ1b-antibody were also negative, and there was no albuminocytological dissociation of his cerebrospinal fluid. The nerve conduction velocity revealed severe reduction in compound muscle action potential (CMAP) amplitudes and motor conduction velocities in bilateral median nerves, ulnar nerves, and radial nerves, while the sensory conduction was normal (Additional file 1 A and B). Right ulnar nerve F-waves were absent. Chronic denervation/reinnervation (e.g., motor unit action potentials of increased amplitude and duration, with reduced inference patterns) was observed in three regions on the electromyogram (EMG), including the bilateral extremities and sternocleidomastoid muscles (Additional file 1 C, D and E). And spontaneous activity (positive sharp waves) was recorded from these muscles. Echocardiography and electrocardiogram evaluations did not detect any cardiac abnormalities. Lower limb muscle MRI showed marked involvement of the gastrocnemius muscle at the calf level. There was a strongly increased signal intensity in turbo inversion recovery magnitude (TIRM) sequences, indicating muscular edema. A mild increase in the signal intensity of soleus and tibialis anterior muscles was observed in the T2 sequence, indicating fat replacement (Fig. 2a and b). At the proximal leg level, slight fatty degeneration was detected in the posterior compartment, such as the semimembranosus and semitendinosus muscles (Fig. 2c and d).

**Fig. 3:**
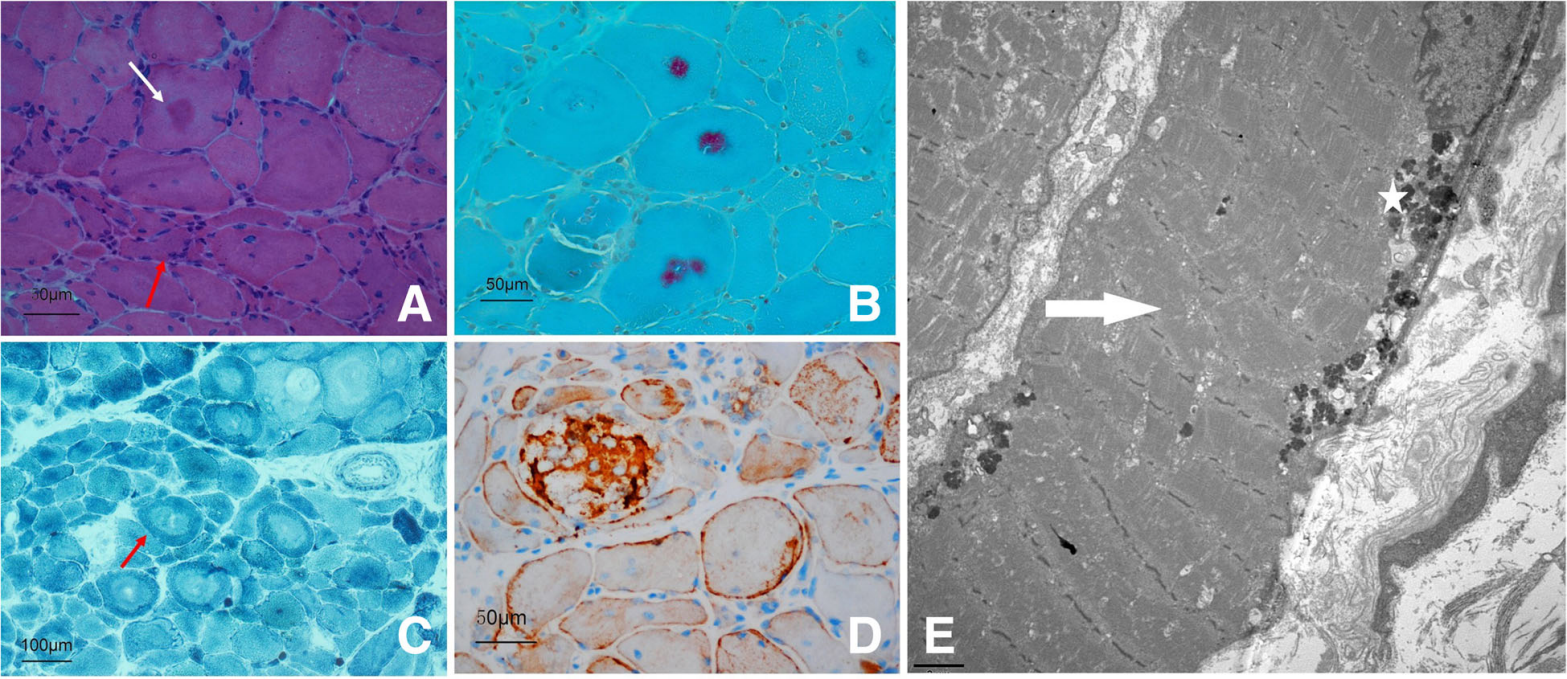
Histopathological examination of the skeletal muscles. **a**: HE staining showing muscle fibers of variable sizes, abnormal material deposits (white arrow), and angular atrophic fibers (red arrow). **b**: MGT staining showing purple myofibrillary inclusions. **c**: NADH-TR staining showing reduced oxidative enzyme activities in some fibers, visible as core-like lesions (red arrow). **d**: Immunohistochemistry staining with an anti-FLNC antibody revealing immunoreactive deposits at both subsarcolemmal and sarcoplasmic levels. **e**: Ultrastructural analysis of a muscle fiber showing myofibrillar disorganization (arrows) and submuscular lipofuscin deposition (star). Magnification, × 5000

After providing written consent, a skeletal muscle biopsy was taken from the patient’s gastrocnemius muscle, precooled with isopentane, and frozen in liquid nitrogen. Frozen sections of 8 μm were then prepared and examined by light microscopy. A marked variation in fiber size was observed, with many angular atrophic fibers. Some fibers also showed structural changes with abnormal material deposits after staining with hematoxylin–eosin (Fig. 3a). On Gomori trichrome-stained sections, these abnormal deposits appeared as purple inclusions. They varied in size, shape, and thickness, and were either single or multiple (Fig. 3b). In the NADH-tetrazolium reductase reaction, oxidative activity was reduced in fibrous areas occupied by the inclusions, showing core-like lesions (Fig. 3c). Neurogenic changes, such as the grouping of angular atrophic fibers, were also present. Immunohistochemical analysis showed prominent FLNC immunoreactive deposits accumulating at subsarcolemmal and sarcoplasmic levels (Fig. 3d). Electron microscopy of the available transverse sections showed an inordinate myofibrillar structure and dissolved myofilaments. Subsarcolemmal accumulations of lipofuscin were also present (Fig. 3e).

**Fig. 4:**
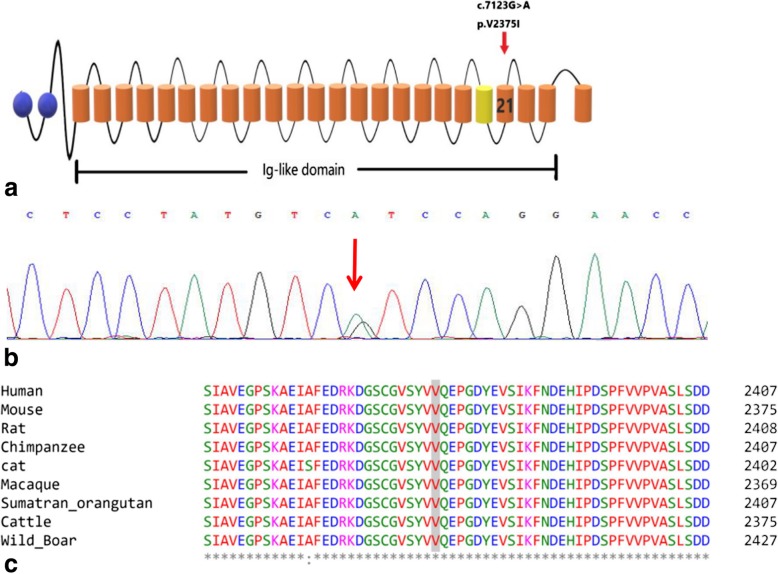
Molecular consequences of the *FLNC* c.7123G > A, p.V2375I mutation. The mutation is located in the 21st Ig-like repeated domain (**a**). DNA sequencing analysis showed a heterozygous missense mutation (c.7123G > A) in *FLNC* (red arrows) (**b**). Partial amino acid sequence alignment of the 21st Ig-like repeat domain of filamin proteins from various species (**c**)

Next-generation sequencing identified a heterozygous missense mutation (c.7123G > A, p.V2375I) in the Ig-like domain 21 of FLNC (Fig. 4a and b). Confirmation of the variant was undertaken by Sanger sequencing using an ABI 3730XL DNA Sequencer (Applied Biosystems, Thermo Fisher Scientific, USA). The mutation was absent in the DNA of 100 healthy unrelated controls, and the allele frequency in the Asian population is zero according to the Exome Aggregation Consortium (http://exac.broadinstitute.org/). The p.V2375I missense mutation affects valine at position 2375, which is highly conserved from mice to humans (Fig. 4c). To exclude other hereditary diseases similar to LMN disease, we also tested for mutations in the genes disrupted in SMAs and the androgen receptor gene, but none were found. Since the patient had no immediate family members and loses contact with other family members, further co-segregation analyses among the family cannot be conducted.

## Discussion and conclusions

MFMs are rare, hereditary and progressive muscle disorders with clinically and genetically heterogeneous [7]. They are defined by the presence of myofibrillar disorganization commencing at the Z-disk, an accumulation of myofibrillar degradation products, and the ectopic expression of multiple proteins. MFM subtypes are designated according to the affected protein, such as desminopathy, aB-crystallinopathy, Bag3opathy, or filaminopathy [8]. MFM subgroup frequencies vary depending on the cohort, with filaminopathy accounting for around 4% of all MFM patients according to the Mayo clinic cohort [9].

Filaminopathy is caused by *FLNC* mutations. Patients with mutations in the *FLNC* Ig-like domain usually present with weakness in limb-girdle muscles, and typical histological characteristics include focal disintegration of myofibrils and the aggregation of sarcoplasmic proteins in muscle biopsies [3, 10]. Another MFM type is caused by missense mutations in the actin binding domain of *FLNC,* which leads to increased actin binding affinity. These patients were described as having a distal myopathy without typical pathological features of MFMs. A frameshift mutation in the Ig-like domain, resulting in haploinsufficiency in *FLNC*, was also shown to be causative of distal myopathy [11, 12].

Patients presenting with distal filaminopathy usually onset at the age of 30–40 years. The most common onset symptom is weakness of the upper limbs, with distal extensors typically more severely affected than flexor muscles. Atrophy is seen mostly in interosseus muscles, especially the first dorsal, and asymmetry is noted in about one-third of patients [13, 14]. Our patient shared the same characteristics. A previously reported Australian family including nine affected individuals with distal filaminopathy showed slow progression of muscle weakness. Lower extremities were affected around 10 years after disease onset. Progression to the proximal muscles was evident in the fifth decade of life and patients usually required a stick for walking during their sixth decade [11]. Our patient showed a more severe clinical pattern. His lower extremities were affected around 6 months after disease onset, and his ambulation is currently limited. This may have been caused not only by muscular lesions, but also by the involvement of peripheral nerves and motor neurons in the spinal cord that presented as LMN syndromes.

LMN syndromes are clinically characterized by muscle atrophy, weakness, and hyporeflexia without sensory involvement. They may arise from disease processes affecting the anterior horn cell or the motor axon and/or its surrounding myelin. Neurophysiological analysis may help support their diagnosis [7, 15]. Our patient’s nerve conduction velocity revealed obviously diminished CMAP amplitudes in the motor nerves, and absent right ulnar nerve F-waves. The presence of chronic denervation was confirmed by long duration, large amplitude, and decreased motor unit recruitment on the EMG. This was observed in the cervical, thoracic, and lumbosacral spinal cord regions, which may indicate that the roots and branches of peripheral nerves, even motor neurons, were damaged. Such neurophysiological findings have not been reported in previous cases of distal filaminopathy, which mainly showed decreased CMAP amplitudes and a myogenic pattern on EMG. Only one patient with distal filaminopathy had mixed myogenic and neurogenic changes in the anterior tibial muscle [11, 13, 14]. While obvious neuropathic changes on EMG can be observed in MFM patients, genetic testing is nevertheless important to help make a correct diagnosis.

LMN syndromes can be hereditary, sporadic, or immune-mediated, and include multifocal motor neuropathy, chronic inflammatory demyelinating polyneuropathy, SMAs, Kennedy’s disease, distal hereditary motor neuropathies, and motor neuron disease [6, 15]. To carry out a differential diagnosis in our patient, we tested for the presence of peripheral neuropathy antibodies, SMA genes, and the androgen receptor gene, but all were negative. Therefore, we suggest that his LMN syndrome is associated with a *FLNC* mutation.

Peripheral nerve involvement has been reported in some MFM patients, including those with desminopathy and ZASPopathy. Moreover, axonal spheroidal formations in the spinal cord and spinal roots that were immunoreactive for neurofilaments were detected in a postmortem of a desminopathy patient [1, 16]. In a German filaminopathy cohort, a muscle biopsy of a patient with hyperesthesia indicated neurogenic changes although nerve conduction velocities were normal [4]. From our current investigation, we suggest that the peripheral nervous system and even the spinal cord could be involved in filaminopathy, with some patients showing a more severe clinical pattern. However, details of the pathological changes and underlying mechanism of filaminopathy should be confirmed by additional studies such as nerve biopsies.

The MRI of our patient disclosed a markedly increased signal intensity in the bilateral gastrocnemius muscles in TIRM sequences, while only a minor increase was seen in T2 sequences. This suggested that the affected muscles had edema, and that they had deteriorated rapidly. Patients in other studies with longer term courses and slower progress had markedly increased signal intensities in T2, consistent with fat replacement and lack of hyperintensity on TIRM sequences indicating an absence of muscular edema. The distribution of affected muscles in our patient was typical. Muscle imaging of filaminopathy often shows a homogenous pattern of muscle involvement. In patients with proximal muscle weakness, the involvement of the vastus intermedius and medialis, adductor magnus, and semimembranosus and biceps femoris muscles is more pronounced. The tibialis anterior muscles, as well as the gastrocnemius and soleus muscles, are usually involved in patients with distal muscle weakness [3, 4, 10–13, 17].

*FLNC* is located on chromosome 7q32-q35 and contains 48 coding exons. Mutation c.7123G > A, p.V2375I in our patient is in the 21st Ig-like domain. Most patients with mutations in *FLNC* Ig-like domains present with weakness in their proximal muscles and have typical protein aggregation in muscle biopsies, while those with mutations in actin-binding domains present with distal myopathy without typical pathological changes on histopathological examination [13, 14]. However, our patient showed the opposite presentation. Despite carrying a mutation in the Ig-like domain, he showed distal myopathy and had typical protein aggregation on muscle biopsy with the presence of large inclusions in the muscle fibers. This suggests that mutations in actin-binding and Ig-like domains cause overlapping clinical symptoms and histopathological changes, but the relationship between genotype and phenotype remains unclear and may be affected by as yet unknown genetic and environmental causes [12].

In conclusion, we report a middle-aged Chinese male with a heterozygous missense mutation (c.7123G > A, p.V2375I) in the Ig-like domain 21 of *FLNC*. He showed distal myopathy and lower motor neuron syndrome with a more severe clinical pattern than other reported cases. Obvious muscle edema could be observed on MRI, and typical protein aggregation presented in the pathological study. Our study not only expands the clinical spectrum of filaminopathies, but also extends the histopathologic and genetic heterogeneity of hereditary distal myopathies and lower motor neuron syndromes. Nevertheless, underlying mechanisms and the relationship between the phenotype and genotype need further study.

## Additional file

Additional file 1: Nerve conduction studies and electromyography results. The nerve conduction velocity revealed severe reduction in CMAP amplitudes and motor conduction velocities in the left ulnar nerve (A), while the sensory conduction was normal(B). Motor unit action potentials of increased amplitude and duration was observed in the EMG, including the right sternocleidomastoid (C), left biceps brachii (D) and left vastus medialis (E). (JPG 166 kb)

## Data Availability

All data generated or analyzed during this study are included in this published article.

## Abbreviations

CMAP: Compound muscle action potential
EMG: Electromyogram
FLNC: Filamin C
LMN: Lower motor neuron
MRI: Magnetic resonance imaging
SMAs: Spinal muscular atrophies
TIRM: Turbo inversion recovery magnitude

## References

[1] Olivé M, Kley RA, Goldfarb LG (2013). Myofibrillar myopathies: new developments. Curr Opin Neurol.

[2] Begay Rene L., Graw Sharon L., Sinagra Gianfranco, Asimaki Angeliki, Rowland Teisha J., Slavov Dobromir B., Gowan Katherine, Jones Kenneth L., Brun Francesca, Merlo Marco, Miani Daniela, Sweet Mary, Devaraj Kalpana, Wartchow Eric P., Gigli Marta, Puggia Ilaria, Salcedo Ernesto E., Garrity Deborah M., Ambardekar Amrut V., Buttrick Peter, Reece T. Brett, Bristow Michael R., Saffitz Jeffrey E., Mestroni Luisa, Taylor Matthew R.G. (2018). Filamin C Truncation Mutations Are Associated With Arrhythmogenic Dilated Cardiomyopathy and Changes in the Cell–Cell Adhesion Structures. JACC: Clinical Electrophysiology.

[3] Kley RA, Serdaroglu-Oflazer P, Leber Y, Odgerel Z, van der Ven PFM, Olivé M (2012). Pathophysiology of protein aggregation and extended phenotyping in filaminopathy. Brain..

[4] Kley RA, Hellenbroich Y, van der Ven PF, Fürst DO, Huebner A, Bruchertseifer V (2007). Clinical and morphological phenotype of the filamin myopathy: a study of 31 German patients. Brain..

[5] Tasca G, Odgerel Z, Monforte M, Aurino S, Clarke NF, Waddell LB (2012). Novel FLNC mutation in a patient with myofibrillar myopathy in combination with late-onset cerebellar ataxia. Muscle Nerve.

[6] Garg N, Park SB, Vucic S, Yiannikas C, Spies J, Howells J (2017). Differentiating lower motor neuron syndromes. J Neurol Neurosurg Psychiatry.

[7] De Bleecker JL, Engel AG, Ertl BB (1996). Myofibrillar myopathy with abnormal foci of desmin positivity. II. Immunocytochemical analysis reveals accumulation of multiple other proteins. J Neuropathol Exp Neurol.

[8] Claeys KG, Fardeau M (2013). Myofibrillar myopathies. Handb Clin Neurol.

[9] Selcen D, Ohno K, Engel AG (2004). Myofibrillar myopathy: clinical, morphological and genetic studies in 63 patients. Brain..

[10] Miao J, Su FF, Liu XM, Wei XJ, Yuan Y, Yu XF (2018). A case report: a heterozygous deletion (2791_2805 del) in exon 18 of the filamin C gene causing filamin C-related myofibrillar myopathies in a Chinese family. BMC Neurol.

[11] Duff RM, Tay V, Hackman P, Ravenscroft G, McLean C, Kennedy P (2011). Mutations in the N-terminal actin-binding domain of filamin C cause a distal myopathy. Am J Hum Genet.

[12] van den Bogaart FJ, Claeys KG, Kley RA, Kusters B, Schrading S, Kamsteeg EJ (2017). Widening the spectrum of filamin-C myopathy: predominantly proximal myopathy due to the p.A193T mutation in the actin-binding domain of FLNC. Neuromuscul Disord.

[13] Guergueltcheva V, Peeters K, Baets J, Ceuterick-de GC, Martin JJ, Suls A (2011). Distal myopathy with upper limb predominance caused by filamin C haploinsufficiency. Neurology..

[14] Rossi D, Palmio J, Evilä A, Galli L, Barone V, Caldwell TA (2017). A novel FLNC frameshift and an OBSCN variant in a family with distal muscular dystrophy. PLoS One.

[15] Verschueren A (2017). Motor neuropathies and lower motor neuron syndromes. Rev Neurol (Paris).

[16] Ariza A, Coll J, Fernández-Figueras MT, López MD, Mate JL, García O (1995). Desmin myopathy: a multisystem disorder involving skeletal, cardiac, and smooth muscle. Hum Pathol.

[17] Avila-Smirnow D, Gueneau L, Batonnet-Pichon S, Delort F, Bécane HM, Claeys K (2016). Cardiac arrhythmia and late-onset muscle weakness caused by a myofibrillar myopathy with unusual histopathological features due to a novel missense mutation in FLNC. Rev Neurol (Paris).

