# Supplementary figures and images for: A mutation in the filamin c gene causes myofibrillar myopathy with lower motor neuron syndrome: a case report
Source: BMC Neurol. 2019 Aug 17;19:198. doi: 10.1186/s12883-019-1410-7 (PMC6697925; doi:10.1186/s12883-019-1410-7)

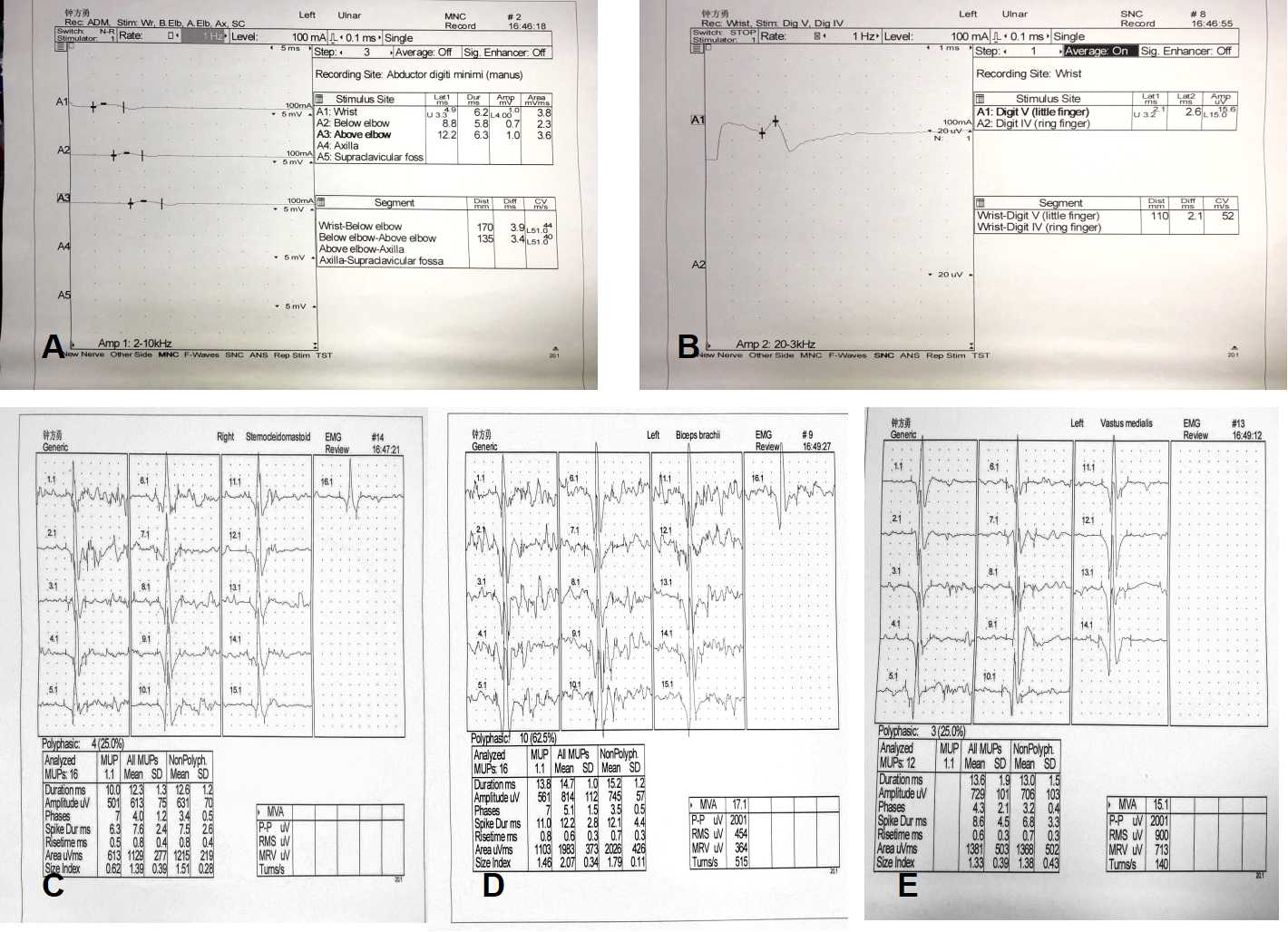

Supplement: Supplementary file 1 — Nerve conduction studies and electromyography results. The nerve conduction velocity revealed severe reduction in CMAP amplitudes and motor conduction velocities in the left ulnar nerve (A), while the sensory conduction was normal(B). Motor unit action potentials of increased amplitude and duration was observed in the EMG, including the right sternocleidomastoid (C), left biceps brachii (D) and left vastus medialis (E). (JPG 166 kb) [file 12883_2019_1410_MOESM1_ESM.jpg]
